# Evaluation of loop-mediated isothermal amplification (LAMP) assay for detection of *aprV2* positive *Dichelobacter nodosus* in-field by secondary users

**DOI:** 10.1186/s13104-019-4575-7

**Published:** 2019-08-22

**Authors:** Nickala Best, Brendan Rodoni, Grant Rawlin, Travis Beddoe

**Affiliations:** 1 Department of Animal, Plant and Soil Science and Centre for AgriBioscience (AgriBio), La Trobe University, Bundoora, Melbourne, VIC Australia; 2 Department of Jobs, Precincts and Regions, Centre for AgriBioscience (AgriBio), Victorian Government, Bundoora, Melbourne, VIC Australia

**Keywords:** Footrot, LAMP, Field, *D. nodosus*, Secondary users

## Abstract

**Objective:**

*Dichelobacter nodosus* is the primary aetiological agent of footrot in sheep. Ovine footrot causes considerable economic losses and substantial animal welfare issues in the Australian sheep industry. Current methods for detecting *D. nodosus* are difficult, laborious and time-consuming. Recently, we developed a robust LAMP assay (VDN LAMP) that was able to identify *aprV2* positive *D. nodosus* in-field. A major advantage of LAMP technology is the ability of the assay to be performed by non-specialists with minimal training. We aimed to assess the performance of the VDN LAMP in-field in comparison to a laboratory-based *aprV2/aprB2* rtPCR when used by secondary users after training by the authors.

**Results:**

Two animal health officers (termed secondary users) from Department of Primary Industries and Regions, South Australia (PIRSA) were trained in the use of VDN LAMP, before carrying out in-field testing on several locations in South Australia. The performance of VDN LAMP assay by secondary user 1 was shown to successfully detect 73.91% (*n *= 53) *aprV2* positive samples, while secondary user 2 detected 37.93% (*n *= 30) *aprV2* positive samples. Overall, the ability to identify virulent *D. nodosus* by VDN LAMP by secondary users was mixed for various reasons, however, this could be rectified by additional training and commercial production of the LAMP kits to increase stability. We envisaged in the future VDN LAMP will able to be used by non-specialists to aid control programs.

## Introduction

The causative agent of footrot is *D. nodosus*, a highly contiguous aerotolerant anaerobe that has the ability to digest hoof material [1]. This results in painful lesions and from the soft underlying tissue of the sheep foot that leads to major welfare and economic concerns. Current testing for *D. nodosus* causing ovine footrot relies on time-consuming and labour-intensive laboratory procedures that can take up to 3–4 weeks for results [2]. This inability to identify *D. nodosus* rapidly allows the infection to spread throughout the farm.

We and others have investigated the use of molecular diagnostics for footrot by PCR and rtPCR [3, 4]. These molecular tests mainly discriminate *D. nodosus* based on presence of *aprV2* and *aprB2* encoding for proteases. Strains possessing AprV2 can cause clinically virulent disease, while AprB2 may cause clinically benign disease [5]. Despite the increased speed of these tests, they still require sending samples to a specialised laboratory. We recently developed a pen-side test using loop-mediated isothermal amplification (LAMP) technology to determine if sheep were infected with *aprV2* positive strains of *D. nodosus* which we have termed VDN LAMP [6, 7]. VDN LAMP has the potential to inform local treatment decisions for the management of footrot in real time. This may help to identify infections prior to severe lesion development, leading to early treatment and reduced spread, or to confirm the presence of *D. nodosus* in the lesion.

LAMP assays have several benefits over other molecular tests, as they are in-field, fast, robust and do not require high-level technical expertise to perform [8]. Despite many advantages of LAMP, very few studies have assessed use by non-technical personnel. Here, we have evaluated the performance of VDN LAMP as used by secondary users to detect *aprV2* positive *D. nodosus* from foot swabs collected in-field.

## Main text

### Materials and methods

#### Training

PIRSA animal health staff were given one 4-h workshop on LAMP use in August 2017 by the primary user (NB). The workshop included background information on molecular diagnostics/LAMP and a run through of sample collection, processing, and a LAMP run with freshly collected samples. Materials provided included pre-made kits, a one-page LAMP workflow and a booklet containing detailed workflow instructions. Two PIRSA animal health staff were chosen as final secondary users, neither person has a significant background in laboratory techniques. These secondary users were provided with kits by the primary user and then performed VDN LAMP over the course of usual FR disease investigations.

#### Sample collection and VDN LAMP for *aprV2* positive *D. nodosus* detection

The samples were collected by Department of Primary Industries and Regions, South Australia animal health officers from the interdigital skin of lame sheep as part of routine diagnostic testing. Current clinical foot scores of the sheep feet were recorded and the single highest scored foot was sampled. Two swabs per sampled sheep were collected simultaneously and used as biological duplicates. One swab was used for *aprV2/aprB2* rtPCR and the second for immediate in-field processing with VDN LAMP.

Swabs for *aprV2/aprB2* rtPCR were collected as previously described [3]. Swabs for in-field processing with VDN LAMP were placed into 500 µL alkaline polyethylene glycol, pH 13.0. Swab heads were snapped into the buffer tubes and left in, with collection and processing occurring at ambient temperature. Sample processing and VDN LAMP reactions were carried out as previously described Best et al. [7], with each kit containing the required reagents in volumes appropriate for the processing of eight samples. Purified genomic gDNA from *arpV2* positive *D. nodosus* strain A198 was used as a positive control for each run.

#### rtPCR for detection of *D. nodosus*

Samples for rtPCR had all nucleic acids present extracted and purified as described previously [3]. The presence of *aprV2* and/or *aprB2* in samples were identified using primers, probes and cycling conditions as described by Stäuble et al. [4]. The AgPath-IDTM One-Step RT-PCR Kit (Ambion, Austin, USA) was used as master mix according to manufacturer’s instructions, adapted for 10 µL final volume. Reactions and analysis were carried out on the Mic rtPCR Cycler (Bio Molecular Systems, Queensland, Australia), using auto threshold detection and bulk analysis.

#### Statistical analysis

Sample results were defined as follows, and only samples with complete data collection were used for analysis;

VDN LAMP positive—sample has both a Tp (< 20 min) and Tm (87.7–88.7 °C).

VDN LAMP negative—sample has only a Tp or Tm, or the Tm does not fall within the above range, or no Tp and Tm.

rtPCR *aprV2* positive—has an rtPCR result in the *aprV2* channel with a Ct < 35.

rtPCR *aprV2* negative—has an *aprV2* Ct ≥ 35, is *aprB2* positive, or negative for *D. nodosus*.

Samples with Ct’s above 35 are considered negative due to a lack of clinical relevance (2). The sensitivity (Se) of VDN LAMP is defined as the percentage of VDN LAMP positive samples within rtPCR *aprV2* positive samples, and the specificity (Sp) the percentage of VDN LAMP negative samples within rtPCR *aprV2* negative samples. Se, Sp, NPV and PPV were calculated using GraphPad Prism 6. Run success is defined as the positive control amplifying before 20 min with a Tm between 87.7–88.7 °C and the negative control failing to amplify.

### Results

A total of 83 sheep from 17 farms are included in the pilot study, sampled from October 2017 to February 2018. Secondary user 1 was based in Naracoorte. Samples were processed on-farm or collected and transported back to the office for processing. Secondary user 1 sampled a total of 53 sheep, from 11 farms. A total of 3 runs were unsuccessful, and a sensitivity of 73.91% and specificity of 100% was seen (Table 1). Secondary user 2 was based in Nuriootpa. Samples were processed on the farm, with a total of 30 sheep from 6 properties provided. Only 2 runs were successful, and the overall sensitivity of 37.93% and specificity of 100% was seen (Table 2).

**Table 1:** Individual farm summary of the success of LAMP run, the number of VDN LAMP and rtPCR samples positive for *aprV2*, the sensitivity seen on that farm and the month of collection by secondary user 1

Farm	Run success	VDN LAMP + (n)	rtPCR *aprV2* + (n)	Se (%)	Collected
1	N	4	5	80.00	Oct
2	Y	1	5	20.00	Nov
3	Y	2	3	66.67	Nov
4	N	1	2	50.00	Dec
5	Y	1	3	33.33	Dec
6	Y	5	5	100.00	Dec
7	N	3	5	60.00	Jan
8	Y	3	3	100.00	Jan
9	Y	5	5	100.00	Feb
10	Y	4	5	80.00	Feb
11	Y	5	5	100.00	Feb
Total		34	46	73.91	

Sensitivity	73.91%	95% CI 58.87 to 85.73%			
Specificity	100%	95% CI 59.04 to 100.00%			
Positive predicative value	100%				
Negative predicative value	36.84%	95% CI 26.4 to 48.69%

**Table 2:** Individual farm summary of the success of LAMP run, the number of VDN LAMP and rtPCR samples positive for *aprV2*, the sensitivity seen on that farm and the month of collection by secondary user 2

Farm	Run success	VDN LAMP + (n)	rtPCR *aprV2* + (n)	Se (%)	Collected
1	Y	4	5	64.00	Oct
2	N	0	5	0.00	Oct
3	N	0	4	0.00	Oct
4	Y	0	5	0.00	Nov
5	N	4	5	64.00	Dec
6	N	3	5	48.00	Dec
Total		11	29	37.93	

Sensitivity	37.93%	95% CI 20.69 to 57.74%			
Specificity	100%	95% CI 02.50 to 100.00%			
Positive predicative value	100%				
Negative predicative value	05.26%	95% CI 04.01 to 06.88%			

Data from secondary user 1 and 2 were also analysed together to provide an overall data set. Combining data shows a sensitivity of 60%, and specificity of 100%, with 46/75 rtPCR *aprV2* positive samples identified in-field by LAMP and secondary users (Table 3). When all samples were grouped based on clinical score, sensitivity was highest in those feet scored 4 (80%), 5 (71.43%), and lowest in those scored 2 (38%) and 3 (46.15%) (Additional file 1: Table S1).

**Table 3:** Agreement of *aprV2* designation between VDN LAMP and rtPCR for all secondary users

	rtPCR *aprV2 *+	rtPCR *aprV2 *−	Total
VDN LAMP +	45	0	45
VDN LAMP −	30	8	38
Total	75	8	83

Sensitivity	60.00%	95% CI 48.04 to 71.15%	
Specificity	100%	95% CI 81.47 to 100.00%	
Positive predicative value	100%		
Negative predicative value	21.05%	95% CI 16.81 to 26.03%	

### Discussion

We have examined the overall performance of the VDN LAMP assay to detect virulent *D. nodosus* when used by non-specialist. VDN LAMP, in the hands of secondary users, showed 60% sensitivity, and 100% specificity. This is in comparison to a sensitivity of 89% and specific of 97%, under ideal conditions, in a study performed by the primary users [6]. Some of the major issues associated with decreased sensitivity of the VDN LAMP assay was the quality control of reagents, contamination and thresholds hold settings. There were instances of delays in transit of the prepared reagent kits between Melbourne and both locations in South Australia. This resulted in incorrect storage of reagents and subsequent poor performance, in some instances failure of controls and therefore VDN LAMP run. This issue may be resolved by the commercialisation of the test, and the creation of dried reagents/kits suitable for transport at ambient temperature. There was also some recorded variation in performance of positive control. This again may be due to variations in delivery times and conditions, physical addition of the sample, or temperature fluctuations. The positive control should ideally amplify in under 13 min, however, was in some instances amplifying later, indicating degradation. It may be possible to include an internal positive control that is less susceptible to degradation, and fluctuations in transport conditions or mechanical errors.

There was one instance of contamination where the negative control amplified. It was difficult to identify the source, so all reagents were disposed of, and equipment bleached. New reagents and bleaching appeared to remedy the situation. As the secondary users are not specialists, additional training of users to decrease the risk of contamination and the development of dried kits that require less handling could help in mitigating the contamination risk.

Additional training could also include data collection, or improvements upon the form used for this study, as sometimes the information provided was unclear. Streamlining the data collection process to create a single location for all information may be of benefit to improving ease of use and clarity of information. This may be through additional labelling on LAMP machine, such as sample labels to include sample information (such as score) that can be read directly on LAMP run files, development of software that integrates assistance by contacting “primary user” and LAMP machine, or integration of data collection into existing frameworks.

Automatic thresholds as set on the GenieIII software were used throughout the study. When looking at the LAMP data files, some annealing peaks are present but are not above the pre-set threshold and so are not included as VDN LAMP positive. This was most apparent in secondary user 2 data. Five additional samples would be VDN LAMP positive with the lowering of the pre-set threshold. A lowering of threshold would increase sensitivity but may also increase false positives. Pre-set thresholds have been appropriate in other VDN LAMP studies and would need to be considered in the context of the intended application of the assay by secondary users.

These results give a greater insight into the use of LAMP technology by non-specialist with clear routes to improving performance with secondary users. This information will help in the deployment of VDN LAMP assay and other diagnostics-based LAMP technology by various Australian Government agencies.

## Limitations

The limitations of this study include a small sample size of sheep, limited training of staff, and less than ideal transport conditions for reagents.

## Supplementary information

**Additional file 1: Table S1.** VDN LAMP agreement to rtPCR *aprV2* designation within clinical scoring groups for footrot for all 83 sheep.

## Data Availability

The information supporting the conclusions of this article is included in the article.

## Abbreviations

rtPCR: quantitative polymerase chain reaction
Tp: time to positive
Tm: annealing temperature
LAMP: loop-mediated isothermal amplification
PIRSA: Department of Primary Industries and Regions, South Australia
