# Supplementary material for: Evaluation of loop-mediated isothermal amplification (LAMP) assay for detection of aprV2 positive Dichelobacter nodosus in-field by secondary users
Source: BMC Res Notes. 2019 Aug 22;12:534. doi: 10.1186/s13104-019-4575-7 (PMC6704695; doi:10.1186/s13104-019-4575-7)
Supplement: Supplementary file 1 — Additional file 1: Table S1. VDN LAMP agreement to rtPCR aprV2 designation within clinical scoring groups for footrot for all 83 sheep. [file 13104_2019_4575_MOESM1_ESM.docx]

Table S1. **VDN LAMP agreement to rtPCR *aprV2* designation within clinical scoring groups for footrot for all 83 sheep.**

| Score | Sheep (n) | VDN LAMP + (n) | rtPCR *aprV2* + (n) | Se (%) |
| --- | --- | --- | --- | --- |
| 1 | 5 | 2 | 4 | 50.00 |
| 2 | 24 | 8 | 21 | 38.10 |
| 3^1^ | 14 | 6 | 13 | 46.15 |
| 4 | 30 | 24 | 30 | 80.00 |
| 5 | 7 | 5 | 7 | 71.43 |
| *Total* | *80^2^* | *45* | *75* | *60.00* |

^1^3A, 3B and 3C all counted as score ‘3’

^2^Three sheep were not scored but noted as ‘abscess’ or ‘T3’
